# Genetic regulation of shoot architecture in cucumber

**DOI:** 10.1038/s41438-021-00577-0

**Published:** 2021-07-01

**Authors:** Xiaofeng Liu, Jiacai Chen, Xiaolan Zhang

**Affiliations:** State Key Laboratories of Agrobiotechnology, Beijing Key Laboratory of Growth and Developmental Regulation for Protected Vegetable Crops, College of Horticulture, China Agricultural University, Beijing, 100193 China

**Keywords:** Plant morphogenesis, Developmental biology

## Abstract

Cucumber (*Cucumis sativus* L.) is an important vegetable crop species with great economic value. Shoot architecture determines the visual appearance of plants and has a strong impact on crop management and yield. Unlike most model plant species, cucumber undergoes vegetative growth and reproductive growth simultaneously, in which leaves are produced from the shoot apical meristem and flowers are generated from leaf axils, during the majority of its life, a feature representative of the Cucurbitaceae family. Despite substantial advances achieved in understanding the regulation of plant form in *Arabidopsis thaliana*, rice, and maize, our understanding of the mechanisms controlling shoot architecture in Cucurbitaceae crop species is still limited. In this review, we focus on recent progress on elucidating the genetic regulatory pathways underlying the determinant/indeterminant growth habit, leaf shape, branch outgrowth, tendril identity, and vine length determination in cucumber. We also discuss the potential of applying biotechnology tools and resources for the generation of ideal plant types with desired architectural features to improve cucumber productivity and cultivation efficiency.

## Introduction

**Fig. 1:**
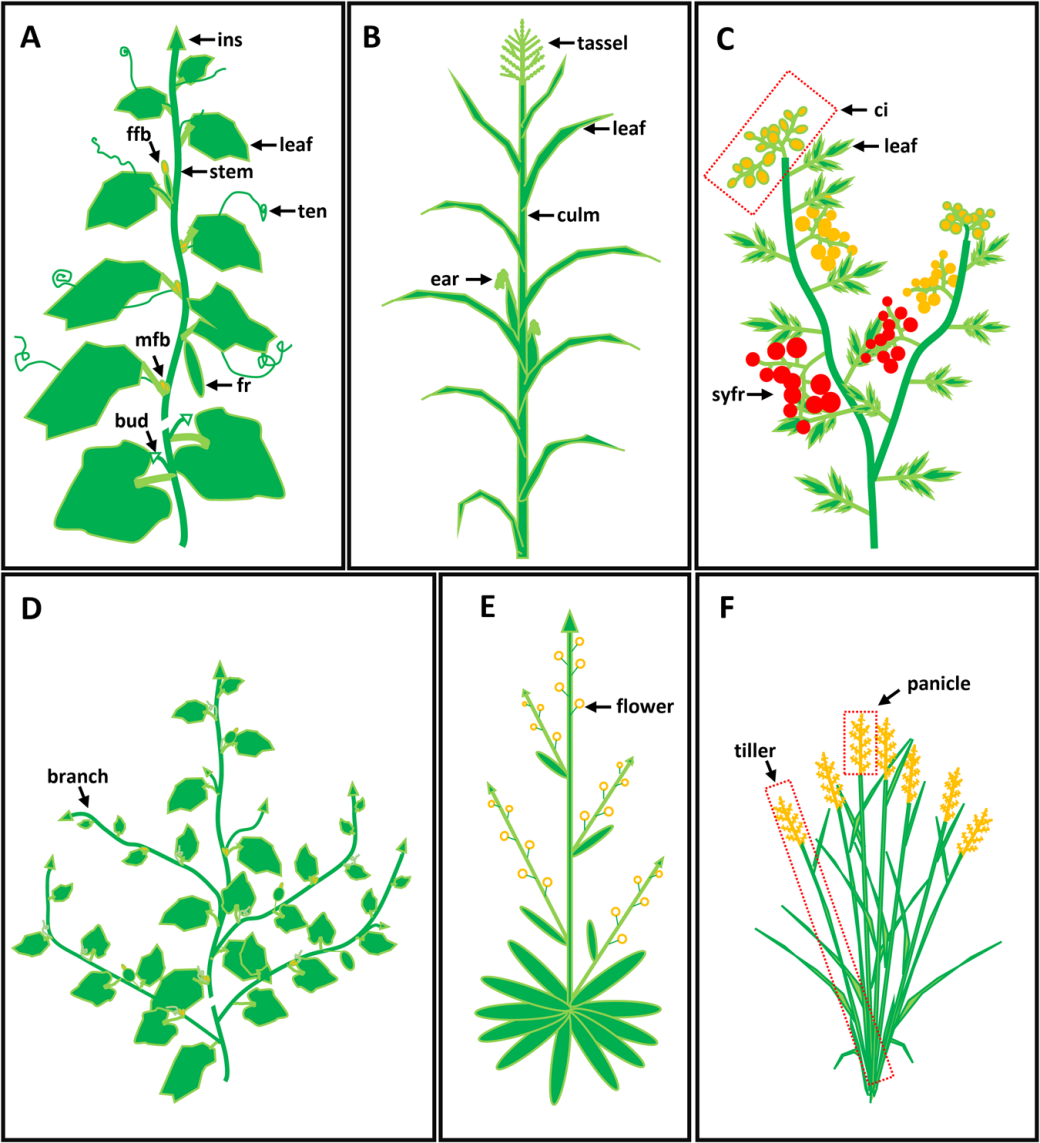
Shoot architecture of cucumber, maize, tomato, *Arabidopsis*, and rice. Representations of shoot architecture of cultivated: **A** cucumber, **B** maize, **C** tomato, **D** wild cucumber, **E** *Arabidopsis thaliana*, and **F** rice. ins, indeterminate shoot tip; ffb, female flower bud; ten, tendril; mfb, male flower bud; fr, fruit; ci, compound inflorescence; syfr, sympodial fruit

In nature, much of the diversity and beauty observed can be attributed to the tremendous variability in plant architecture. Despite its plasticity to prevailing environmental conditions, shoot architecture has species-specific characteristics, indicating the involvement of genetic regulatory mechanisms. In flowering plants, shoot architecture is largely determined by the organization and activities of meristems, including apical, axillary, and inflorescence meristems^1^. During the vegetative stage, the shoot apical meristem (SAM), located at the shoot tip, produces leaf primordia from its end in a sequential and modular manner, and vegetative shoots are generated from axillary meristems in leaf axils^2,3^. Upon transition to the reproductive phase, the SAM turns into an inflorescence meristem (IM) that produces flowers directly or flower-bearing shoots^4–6^. In most plant species, such as *Arabidopsis thaliana* and rice, the transition from vegetative to reproductive growth is easy to discern. Cucumber (*Cucumis sativus* L.), an important vegetable crop species in the Cucurbitaceae family, undergoes vegetative growth and reproductive growth simultaneously during the majority of its life, in which leaves are produced from the SAM, while flowers, branches, and tendrils are produced from the leaf axils^7–9^. Therefore, the development of stems, leaves, tendrils, branches, flowers, and fruits determines the shoot architecture of cucumber (Fig. 1A).

Shoot architecture has a strong impact on crop management and yield and thus has been subjected to intense selection during crop domestication and improvement. For example, modern maize has single-culm-bearing ears at the axillary nodes and tassels at the shoot tip (Fig. 1B), whereas its ancestor, teosinte, is highly branched^10^. Tomato is a model species of sympodial plants that produce compound inflorescences (Fig. 1C); during domestication, its fruit size increased, and its fruit shape became diversified^11,12^. Similarly, the first Green Revolution resulted in a significant increase in rice yield, mainly due to the rapid adoption of semidwarf cultivars^13^.

Cucumber, an annual creeping or climbing crop species with unisexual flowers, has a 90~120 day life cycle. Based on nucleotide diversity, cucumber can be divided into four geographic groups: an Indian group, a Xishuangbanna group, a Eurasian group, and an East Asian group^7^. Compared to the wild ancestor *C. sativus* var. *hardwickii* in the Indian group, commercial cucumbers have reduced branches, stronger stems, increased leaf size, and enlarged fruits (Fig. 1A, D). Suitable plant architecture can improve crop yield and reduce labor costs, which is of great importance to feed the increasing population given the limited arable land available. Identifying the key genes and molecular mechanisms controlling shoot architecture is essential for the efficient modification of plant forms with desired architectural traits. In this review, we summarize the recent progress on elucidating the genetic regulatory pathways underlying determinant/indeterminant growth, leaf shape, branch outgrowth, tendril identity, and vine length determination in cucumber. We also describe the potential of applying biotechnology tools and resources to further improve production and cultivation efficiency by breeding ideal shoot architecture into cucumber.

## Cucumber has both indeterminate and determinate growth habits

Plants can be divided into two groups based on whether the primary inflorescence axis turns into flowers at the end or not: those that have determinate growth and those that have indeterminate growth. *Arabidopsis* undergoes indeterminate growth, as its flowers and secondary inflorescence branches are produced from the main stem (Fig. 1E)^1^, whereas rice undergoes determinate growth, as the primary IM terminates into a spikelet and floral meristems (Fig. 1F)^14^. In cucumber, vegetative growth and reproductive growth occur simultaneously; thus, it is the SAM, not the IM, which specifies the indeterminate/determinate growth habits^15^. Most cucumber varieties grown for fresh markets have an indeterminant growth habit, which is advantageous for continuous harvesting when grown in protected environments to obtain high yields, and are adapted for manual harvest^16^. In contrast, cucumber varieties used in the pickling industry generally undergo determinate growth, which is desirable for mechanical harvesting and high-density planting in open fields^17,18^.

Indeterminate/determinate growth habit is driven by the balance between IM identify and floral meristem identity, which is regulated by interplay between TERMINAL FLOWER 1 (TFL1), LEAFY (LFY), and APETALA1 (AP1)^19–22^. The *AP1* and *LFY* genes are crucial for specifying floral meristem identity in *Arabidopsis*. *AP1* and *LFY* encode MADS box and plant-specific transcription factors, respectively, which are expressed in the floral meristem. Loss-of-function of *AP1* and *LFY* results in the replacement of initial flowers on inflorescence stems with shoots^19,23^. In *Arabidopsis*, *LFY* and *AP1* activate the expression of each other through a positive feedback loop^21^. TFL1 acts as a key player repressing floral induction and maintaining shoot indeterminacy^24,25^. *Arabidopsis tfl1* mutants display determinate growth, with the conversion of the IM into a terminal flower^24^. *TFL1* encodes a phosphatidyl ethanolamine-binding protein (PEBP) and is expressed in the central region of the IM; TFL1 represses *AP1* and *LFY* expression in the IM^22,25,26^. In turn, AP1 suppresses *TFL1* expression, and LFY promotes the expression of *TFL1*^19,21^. The mutual regulatory network involving AP1, LFY, and TFL1 shows the complexity underlying floral initiation. SELF-PRUNING (SP), the tomato ortholog of TFL1, has conserved functions in repressing floral induction and maintaining indeterminate growth^27^. The spontaneous loss-of-function *sp* mutant exhibits a series of desired characteristics for mechanical harvesting, including determinate growth, reduced plant height, and uniform fruit ripening; therefore, the *sp* allele was selected and introgressed into modern tomato cultivars^27^. Similarly, TFL1 homologs in *Antirrhinum* and rice have conserved functions in inhibiting floral induction and maintaining meristem indeterminacy^28,29^. Another PEBP, FT, together with TWIN SISTER OF FT (TSF), functions antagonistically with TFL1 in specifying IM identity in *Arabidopsis*^30^. SINGLE FLOWER TRUSS (SFT), a FLOWERING LOCUS T (FT) ortholog, and SUPPRESSOR OF SP (SSP), a homolog of *Arabidopsis* FD, were identified as suppressors of *SP* to maintain indeterminate growth in tomato^11,31–33^.

**Fig. 2:**
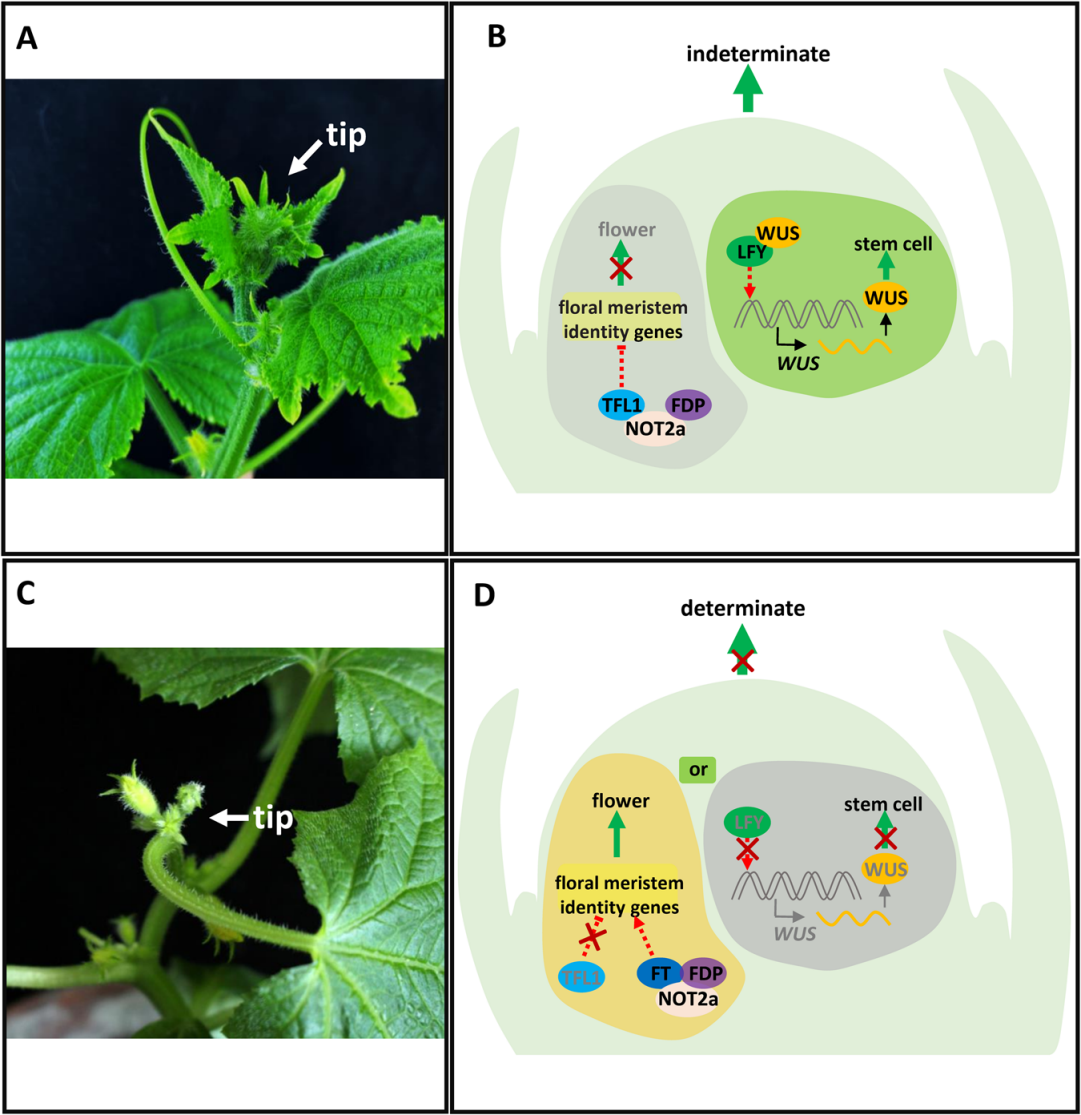
CsLFY and CsTFL1 coregulate the indeterminate/determinate growth habit of cucumber. Cucumber plants with: **A** indeterminate, and **C** determinate growth habits. **B** CsTFL1 promotes indeterminate growth by forming a complex together with CsNOT2a and CsFDP to repress floral meristem development. CsLFY directly interacts with CsWUS in the SAM to maintain stem cell identity and thus maintain an indeterminate growth habit. **D** The absence of CsTFL1 or CsLFY results in a determinate growth habit of cucumber

In cucumber, mapping for the *determinate* (*de*) locus showed that a SNP in *CsTFL1* underlies the determinate growth habit^16^. Knockdown of *CsTFL1* by RNAi led to terminal flowers at the shoot apex, confirming the role of CsTFL1 in regulating indeterminate growth in cucumber^16^. In situ hybridization showed that *CsTFL1* transcript signals were present in the subapical regions of the SAM, lateral meristems, and young stems. Biochemical analyses indicated that CsTFL1 competes with CsFT for interaction with the CsNOT2a (negative on TATA-less 2a)-CsFDP (FD PARALOG) complex to suppress floral meristem identity genes in the shoot tip to promote the indeterminate growth of cucumber (Fig. 2)^16^. *CsLFY* was cloned in cucumber, and knockdown of *CsLFY* resulted in disrupted shoot apex development and premature termination of leaf initiation, suggesting that CsLFY has a novel function in regulating shoot meristem maintenance in cucumber. CsLFY directly interacts with CsWUS (WUSCHEL) in the SAM to maintain stem cell identity and thus maintain an indeterminate growth habit^15^. Therefore, CsTFL1 and CsLFY coordinately regulate the indeterminate growth habit of cucumber by suppressing floral meristem development and promoting stem cell identity in the SAM, respectively (Fig. 2).

In addition, unfavorable environmental conditions can result in the transition from indeterminate growth to determinate growth, referred to as the ‘blunt with blossom’ conditions, during cucumber cultivation. A succession of low-irradiance days, low temperature, and drought are the main factors that give rise to the ‘blunt with blossom’ condition, which is associated with reduced yields of cucumber and decreased cucumber fruit quality^17^. The genetic mechanisms underlying the above environmental factors leading to ‘blunt with blossom’ remain unidentified in cucumber.

## Genetic regulation of leaf morphology in cucumber

Leaves are planar lateral appendages of plants and function as solar panels that capture sunlight, and they are used for carbohydrate and oxygen generation. Leaves also act as the interface for sensing signals of the surrounding environment, including light, temperature, water, insects, and microbes^34^. Therefore, leaf morphology plays important roles in photosynthesis, planting density, crop yield, and cultivation labor cost. Leaves originate from ends of the SAM and develop into planar structures along three axes: the adaxial–abaxial axis, proximal–distal axis, and mediolateral axis^34,35^. Considerable advances have occurred in the understanding of the key genes and phytohormones involved in the regulation of leaf initiation, leaf polarity determination, leaf flattening, and intercalary growth of *Arabidopsis* and tomato^34^.

**Fig. 3:**
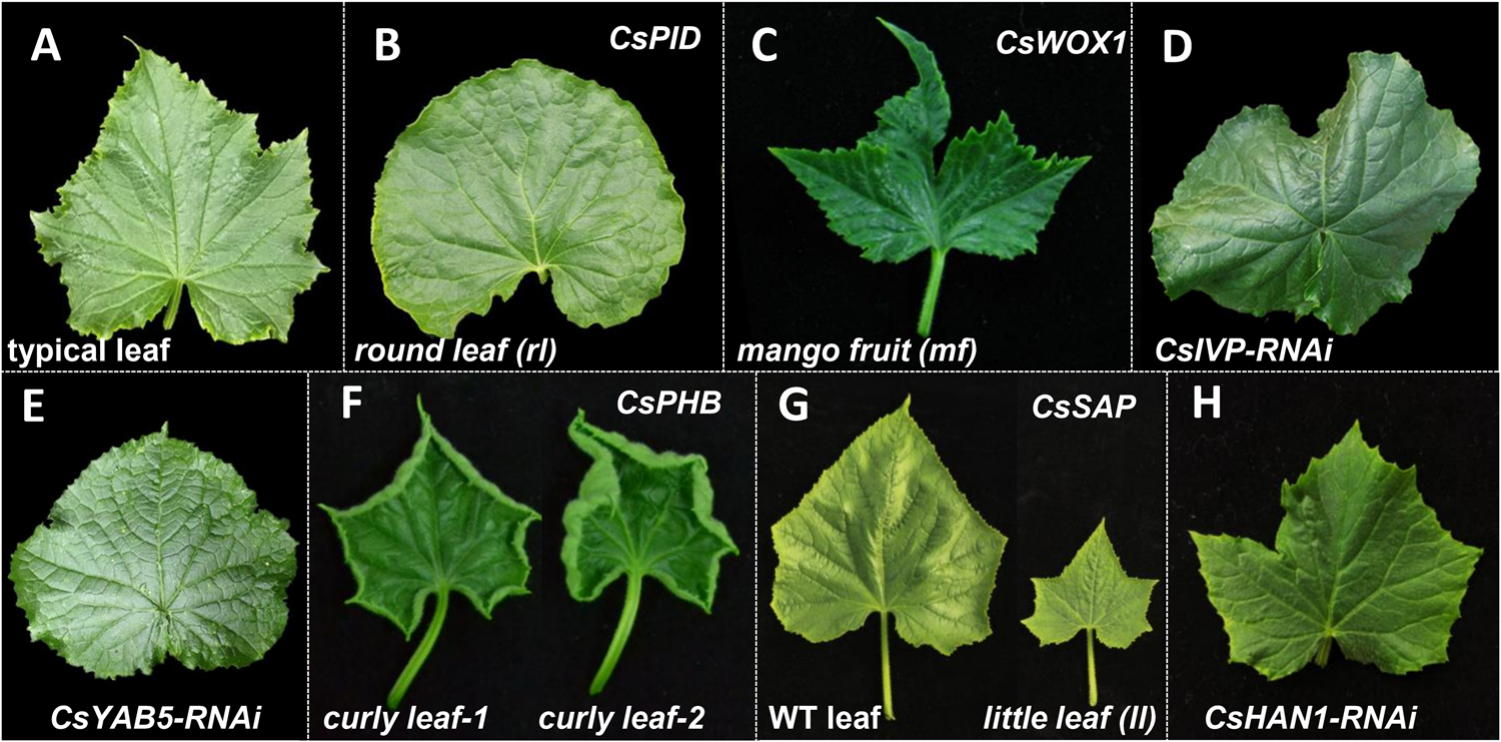
Morphological phenotypes of representative cucumber leaf mutants or transgenic lines. **A** Typical leaf of cucumber, **B** the *round leaf (rl)* mutant, **C** the *mango fruit* (*mf*) mutant, **D** the *CsIVP*-RNAi line, **E** the *CsYAB5*-RNAi line, **F** the *curly leaf-1* (*cl-1*) and *curly leaf-2* (*cl-2*) mutants, **G** the *little leaf* (*ll*) mutant and its WT control, and **H** the *CsHAN1*-RNAi line. The causal genes underlying the phenotype are listed

Cucumber is a typical dicotyledonous plant species that produces simple leaves; in this case, a single leaf blade is attached to the node by a petiole^34,36^. A typical leaf of cucumber is palmate, with five primary veins extending from the petiole at the leaf base to the leaf margins to form lobed leaf (Fig. 3A). In recent years, mutants with abnormal leaf morphology have been identified, and several genes have been mapped and characterized (Fig. 3). In the *round leaf* (*rl*) mutant, the primary leaf vein branches into secondary or higher-order veins to generate a smooth leaf edge, which results in rounded leaves (Fig. 3B). Fine mapping data showed that the causal gene *rl* encodes a homolog of the protein kinase PINOID in *Arabidopsis* (CsPID)^36–38^. PID is involved in the fine-tuning of polar auxin transport through phosphorylation of PIN-FORMED (PIN) proteins in *Arabidopsis*^39^. In cucumber, CsPID regulates the distribution of indoleacetic acid (IAA) in leaves by mediating polar auxin transport, biosynthesis, and signaling pathways to drive leaf vein patterning^37^. A cucumber *mango fruit* (*mf*) mutant with a disrupted WOX1-type protein (CsWOX1) displayed lamina developmental defects and abnormal vein patterning. The *mf* leaves have a butterfly-like shape and substantial growth defects in the mediolateral axis (Fig. 3C)^40,41^. Based on the genetic analysis of the *mf rl* double mutant, CsWOX1 functions in leaf vein patterning via CsPID-mediated auxin transport. Moreover, CsWOX1 regulates leaf size by interacting with CIN (CINCINNATA)-TCP (TEOSINTE BRANCHED1/CYCLOIDEA/PCF) proteins^41^. Two transcription factors, *CsIVP* (*Cucumis sativus Irregular Vasculature Patterning*) and *CsYAB5* (*Cucumis sativus YABBY 5*), are highly expressed in vascular tissues to regulate leaf morphology in cucumber^42^. In *CsIVP-RNAi* plants, the leaves curl downward, and the bilateral leaf margins overlap due to the enlarged primary veins and increased number of secondary veins (Fig. 3D)^42^. Similarly, knockdown of *CsYAB5* by RNAi led to abnormal leaf morphology with overlapping bilateral leaf margins (Fig. 3E). Biochemical analyses have indicated that CsIVP directly binds the promoter of *CsYAB5* to promote its expression to regulate leaf shape in cucumber^42^. The leaves of two gain-of-function mutants, *curly leaf-1* (*cul-1*) and *curly leaf-2* (*cul-2*), roll upward (Fig. 3F). Mapping data showed that the candidate genes underlying *cul-1* and *cul-2* are located within a *cs-miRNA165/166* target sequence of *CsPHB* (*Cucumis sativus PHABULOSA*), a homolog of *Arabidopsis* PHABULOSA, which belongs to the class III homeodomain-leucine zipper (HD-ZIP III) transcription factor family^43^. In *Arabidopsis*, HD-ZIP III transcription factors determine adaxial cell identity in leaf polarity determination, and *AtPHB* gain-of-function mutants resemble the *cur-1* and *cur-2* mutants with upward curling leaves, indicating that the function of PHB is conserved in adaxial–abaxial specification during leaf development^43–46^. In addition, genes controlling cell proliferation and expansion generally also affect organ size^47^. The small-leaf phenotype of the *little leaf* (*ll*) mutant was due to reduced cell numbers and smaller cell size in cucumber (Fig. 3G), and the candidate gene *LL* encodes an F-box protein with multiple WD40 repeats, which is a homolog of *Arabidopsis* SAP (STERILE APETALA)^48^. In the *small and cordate leaf 1* (*scl1*) mutant, the leaf base is blunt, and the leaf size is reduced due to decreased cell numbers^49^. Through bulked segregant analysis-based sequencing (BSA-seq), the causal gene of *scl1* was identified as *Csa7G06276*0, which encodes a putative nucleoside bisphosphate phosphatase belonging to the polymerase and histidinol-phosphatase (PHP) family within the amidohydrolase superfamily^49^. *CsHAN1* (*HANABA TARANU*), encoding a GATA3-type transcription factor, also participates in leaf shape development. The leaves of both *CsHAN1* overexpression and *CsHAN1* knockdown lines are highly lobed, especially those along the first 10 nodes (Fig. 3H)^50^.

## Shoot branching is an important agronomic trait in cucumber

Shoot branching is a fundamental process of plant growth and fitness and is linked to crop yield; thus, shoot branching has been as a selection target during domestication^10,12^. Compared to the wild ancestor of cucumber with many axillary branches, commercial cucumber cultivars have very few branches due to artificial selection. Cucumber plants grown in protected environments for fresh markets, such as Chinese Long, produces fruits along the main stem, which is adapted for continuous harvesting. Axillary branches need to be manually removed to reduce energy consumption and ensure high yields; however, removal of axillary branches increases labor costs. For cucumber plants grown in open fields for the pickling industry, such as GY14, which produces small fruits, stronger lateral branch growth potential is preferred to improve productivity. Therefore, elucidation of the genetic regulatory mechanism of shoot branching is important for breeding cucumber varieties adapted to different cultivation systems.

**Fig. 4:**
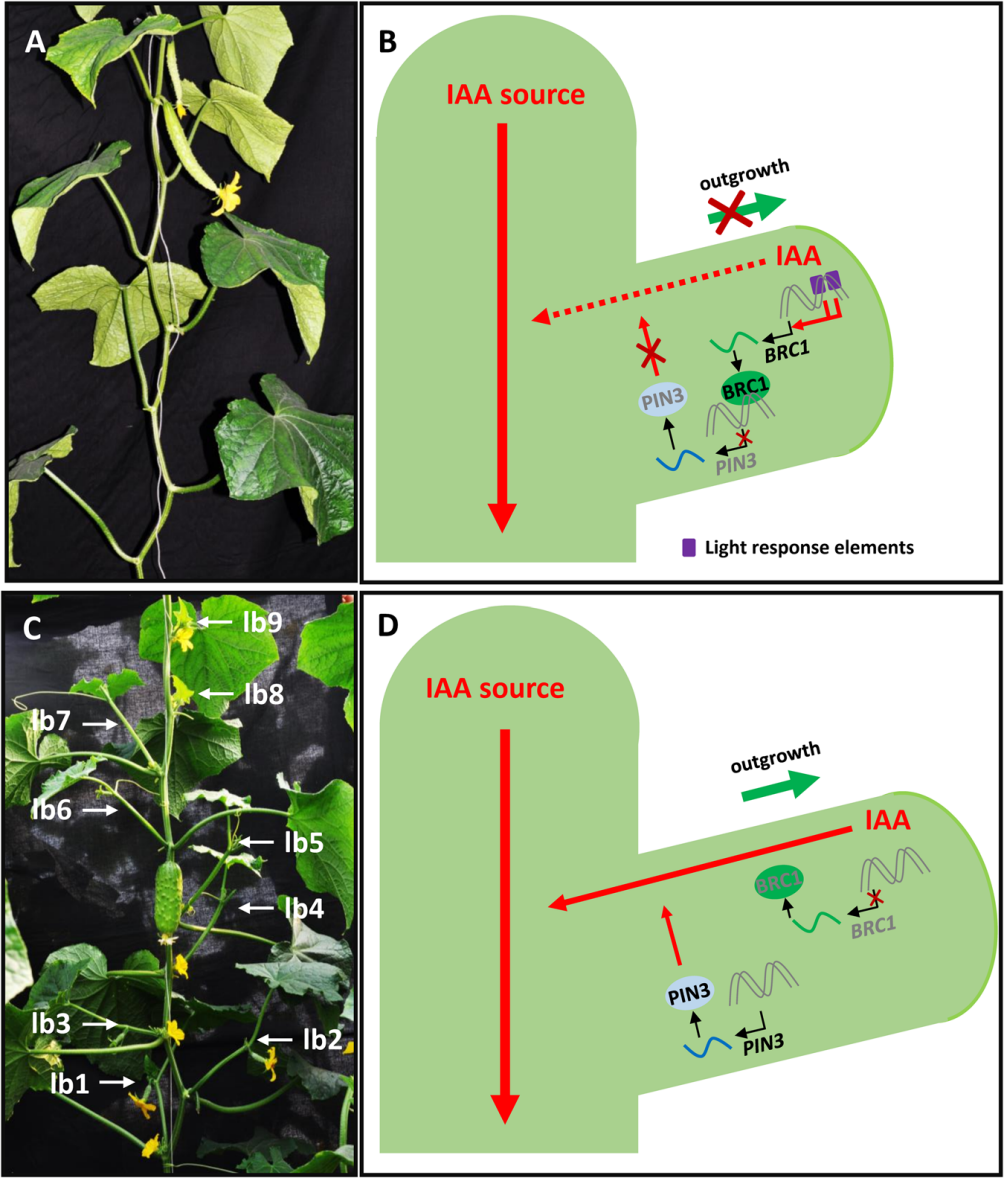
CsBRC1 represses bud outgrowth by directly inhibiting *CsPIN3* activity in cucumber. **A** Representative cucumber plant with no branch outgrowth. **B** CsBRC1 negatively regulates bud outgrowth by directly inhibiting the transcription of the auxin transporter *CsPIN3* and thus auxin accumulation in lateral buds. **C** Representative cucumber plant with many lateral branches. **D** The absence of CsBRC1 leads to increased expression of *CsPIN3*, depleted auxin accumulation in the buds, and lateral bud outgrowth of cucumber. lb: lateral branch

The formation of lateral branches is controlled by axillary bud initiation and subsequent bud outgrowth. *Lateral Suppressor* (*LS*), a GRAS family transcription factor, regulates the formation of the axillary meristem in tomato. In the *ls* mutant, lateral meristems do not form during the vegetative stage^51^. *MONOCULM 1* (*MOC1*) and *LATERAL SUPPRESSOR* (*LAS*), the rice and *Arabidopsis* orthologs of *LS*, respectively, have conserved functions in bud initiation^52,53^. The *Cucumber Lateral Suppressor* (*CLS*) gene has been cloned, and its transcripts accumulate in leaf axils where axillary meristems initiate^54^. Ectopic expression of *CLS* in *Arabidopsis* fully complemented the reduced number of axillary buds of the *las* mutant^54^, indicating the conserved function of CLS in bud initiation in cucumber. Auxin was found to be the primary hormone responsible for apical dominance to repress lateral bud outgrowth^55,56^. The TEOSINTE BRANCHED 1/CYCLOIDEA/PCF (TCP) family gene known as *BRANCHED 1* (*BRC1*) in eudicots and *teosinte BRANCHED 1* (*TB1*) in monocots was shown to be the key factor repressing shoot branching in different species, including *Arabidopsis* (*AtBRC1*), tomato (*SlBRC1b*), rice (*OsTB1*), and maize (*TB1*)^57–61^. In cucumber, *CsBRC1-RNAi* lines exhibit increased shoot branching and reduced auxin accumulation in lateral buds^62^. Biochemical data showed that CsBRC1 inhibits the expression of the auxin efflux carrier *PIN-FORMED* (*CsPIN3*) by directly binding to its promoter. Increased expression of *CsPIN3* driven by the *CsBRC1* promoter resulted in increased numbers of lateral branches and reduced auxin accumulation in the buds^62^; this study provides a direct link between auxin and CsBRC1 in regulating bud outgrowth in cucumber. During domestication, two insertions of light response elements in the *CsBRC1* promoter may have contributed to the increased expression of *CsBRC1* in cultivated cucumber in the adaptation to high-density planting and increased productivity (Fig. 4)^62^.

## Cucumber is a climbing plant due to tendrils

Cucurbitaceous crop species can climb via tendrils, which are specialized organs with a filamentous structure arising from leaf axils. Tendrils provide winding support for plants to arrive at higher or advantageous positions for capturing more sunlight or other beneficial resources^63,64^. Tendrils of cucurbitaceous crop species are modified branches^65^. Tendrils of cucumber and melon are branchless, whereas those of watermelon and pumpkin are ramate tendrils, with 2–4 branches^65,66^. Tendrils can twine around other supportive structure during climbing. First, the initially straight tendrils find an attachment point. Then, the touch-sensitive region near the tendril tip senses a thigmotropic signal and begins to climb the perceived structure within seconds or minutes via twining. Finally, tendrils coil by forming two opposing helices with approximately 10 turns on each side of a perversion point to host the plant shoot toward the attachment point^65,67,68^. Studies have shown that lignified gelatinous fiber ribbons are found on only the ventral side of tendrils, resulting in the ventral side shrinking longitudinally relative to the dorsal side through asymmetric contraction and tendril coiling in cucumber^67^. For cucumber cultivation in protected environments, the climbing capacity of tendrils gives rise to disorderly growth and inconvenient crop management. Therefore, tendrils need to be manually removed in a timely manner, and the growing direction of the main vines is usually specified via artificial hanging, which greatly increases labor costs. Moreover, the growth and coiling of tendrils utilize a considerable portion of plant biomass. As such, tendrillessness is a desirable agronomic trait for cucumber production and breeding.

Among cucumber germplasm resources, tendrillessness or abnormal tendrils are quite rare; only four genes have been identified as being involved in tendril development in cucumber. In the *tendril-less* (*ten*) mutant, tendrils are replaced with branches, and climbing ability of the plant is lost. The causal gene underlying the *ten* mutant is *TENDRIL-LESS* (*TEN*), which encodes a TCP transcription factor expressed specifically in tendrils^67^. Further study showed that the C-terminus and N-terminus of TEN perform different functions to regulate tendril identity and coiling^68^. TEN binds to intragenic enhancers (CDCCRCC motifs) of target genes through the C-terminal domain, whereas its N-terminus functions as a noncanonical histone acetyltransferase to preferentially modify the H3 globular domain; thus, the C- and N-terminus coordinately participate in chromatin loosening and host gene activation^68^. Moreover, ethylene has been found to induce spontaneous tendril coiling, and TEN was shown to be recruited to exons of both *ACC OXIDASE 1* (*ACO1*) and *ETHYLENE RESPONSE FACTOR 1* (*ERF1*) to regulate their transcription. The tendril coiling capacity is substantially altered in *aco-1* and *aco-2* mutant plants^68^. Similarly, CLT, the ortholog of TEN in melon, was also identified as a key regulator determining tendril identity^69^. Another study showed that a histone acetyltransferase encoded by *Cucumis sativus GENERAL CONTROL NONDEREPRESSIBLE 5* (*CsGCN5*) is the candidate gene responsible for the tendril-less mutant *B007*, in which a nonsynonymous SNP in the first exon of *CsGCN5* leads to an amino acid substitution from Asp (D) in the wild-type to Asn (N) in the *B007* mutant^70^, suggesting that histone acetylation status is important for tendril growth and development. In addition to the tendril-less phenotype, *B007* mutant plants exhibit pleiotropic phenotypes, including a smooth epidermis, sterile female flowers, and a dwarf stature^70^. Auxin transport was found to play an essential role in lateral organ morphogenesis. CsPID, whose homolog in *Arabidopsis* regulates auxin transport via phosphorylation of auxin efflux transporters, was shown to control tendril initiation along the first ~20 nodes of cucumber plants^37^. The cucumber *pid* mutant also has decreased numbers of other lateral organs, such as leaves and flowers^37^.

## Vine length is a pivotal factor in cucumber shoot architecture

Grafting cucumber seedlings onto pumpkin (*Cucurbita moschata*) rootstocks is widely used to improve fruit yield and quality during cucumber cultivation^71^. Grafting survival rate is strongly influenced by hypocotyl growth of cucumber seedlings. Environmental conditions such as low light, high temperature, and high humidity generally result in excessive growth of the hypocotyl and thus poor seedling quality for grafting and transplanting^72^. A *short hypocotyl 1* (*sh1*) allele encoding a human SMRCA3-like chromatin remodeling factor is enriched in semiwild Xishuangbanna (*C. sativus* L. var. *xishuangbannesis*) and wild (*C. sativus* L. *var. hardwickii*) cucumber populations. The *sh1* allele allows hypocotyl elongation that is insensitive to UV-B-free light and temperature, which is beneficial for industrialized seedling production of cucumber^72^. The *long hypocotyl* (*lh*) mutant has a saturated shade avoidance response due to the lack of a light-stable PHYB-like phytochrome^73^. On the other hand, hypocotyl elongation tends to be reduced under high light intensity, which is partly due to gibberellin (GA) deactivation^74,75^. The levels of two transcripts of *Gibberellin 2-beta-dioxygenase* (*CsGA2ox8*) are precisely regulated to control plant height under high-light stress. With increasing light intensity, nonfunctional *CsGA2ox8.2* transcripts are generated to buffer against functional *CsGA2ox8.1* transcripts to finely tune GA levels^75^. In addition, a G protein, CsGPA1, was found to positively regulate hypocotyl growth by promoting increased cell size in cucumber overexpression and RNAi-transgenic lines^76^.

In the adult plant stage, suitable compact plant types are preferred in cucumber production for once-over mechanical harvesting and high-density planting^77^. Six mutants with dwarf phenotypes have been identified: *Cucumber dwarf* (*Csdw*), *compact* (*cp*), *compact-1* (*cp-1*), *super compact-1* (*scp-1*), *super compact-2* (*scp-2*), and *short internode* (*si*)^77–81^. The *Csdw*, *cp*, *cp-1*, *scp-1*, and *scp-2* mutants have extremely short internodes and thus little to no practical application value. The length of the internodes of the *Csdw* mutant is reduced because of decreased cell numbers in the main stem and reduced endogenous GA3 levels, which can be partially rescued by GA3 application. MutMap and kompetitive allele-specific PCR (KASP) genotyping results revealed that *CsDW*, which encodes a CLAVATA 1-type receptor-like kinase, is the putative candidate gene for the *Csdw* mutant^78^. Similarly, fine genetic mapping indicated that the *cytokinin oxidase* (*CKX*) and *Cullin 1* genes may be potential candidates for the *cp* and *cp-1* mutants, respectively^77,82^. *CsCYP85A1*, encoding a brassinosteroid (BR)-C6 oxidase involved in BR biosynthesis, was found to be responsible for the extreme dwarfism of *scp-1*, and exogenous application of BR could rescue the mutant phenotype^83^. A spontaneous single-base insertion of *DEETIOLATED-2* (*CsDET2*) led to the supercompact phenotype and systemic BR deficiency of *scp-2*. *CsDET2* encodes a steroid 5-alpha-reductase that acts in the early step in brassinolide synthesis, and exogenous BR treatment could partially rescue the dwarf phenotype of *scp-2*^81^. *CsVFB1*, a member of the VIER F-BOX PROTEIN subfamily of the F-Box protein family, was identified to participate in short internode development in the *si* mutant^79^. Moreover, both *CsIVP-RNAi* plants and *CsYAB5-RNAi* lines displayed a dwarf stature with fasciated vasculature of the stems^42^.

In addition, flowering time, sex determination, and fruit-related traits have some effect on the shoot architecture of cucumber. Two large deletions (‘short-1’ and ‘short-2’) in upstream sequence of *CsFT* (*Flowering Locus T*) were found to be associated with increased *CsFT* expression and advanced flowering and were distributed mostly in populations at relatively high latitudes, suggesting that *CsFT* has undergone positive selection during the domestication of acclimatization of cucumber^83^. Cucumber produces three kinds of flowers: male flowers, female flowers and hermaphroditic flowers. Four genes, *F* (*female*), *M* (*andromonoecious), A* (*androecious*), and *Gy* (*gynoecious*), control cucumber sex determination by regulating ethylene biosynthesis^84^. For fruit-related traits such as fruit shape, fruit color, fruit spine, the presence of fruit warts, and fruit wax, numerous studies have been performed, and specific reviews on cucumber should be consulted for additional details^85^.

## Perspectives

In this work, we summarized 25 genes identified as being involved in shoot architecture traits, including determinate growth habit (2), leaf morphology (8), lateral branching (3), tendril formation (4), vine length (10), and flowering (1); among them, *CsPID*, *CsIVP*, and *CsYAB5* are involved in two developmental processes (Table 1). Most of these have been cloned from natural germplasm resources, obtained from spontaneous mutations, or cloned from EMS-induced mutagenesis populations, and some of them were identified by transgenic analyses (Table 1). Shoot architecture is a holistic and systematic phenotype, and mutations in one gene often cause changes in multiple organs, which is undesirable for fine-control strategies of modern breeding. For example, despite tendrillessness being a desirable feature in the *td-1* mutant, severe dwarfism and sterile flowers are deleterious to yield and thus are associated with decreased value in terms of cucumber breeding^70^. In another *tendril-less* mutant (*ten*), tendrils are replaced only by short branches, and other developmental processes are unaffected^66^. Hence, the *ten* alleles serve as an important genetic resource for designing ideal cucumber architecture. In addition, some well-known genes have potential use in cucumber breeding. For example, the suitable compact shoots of *si* are preferred for once-over mechanical harvesting and high-density planting^79^; *sh1* hypocotyl elongation that is insensitive to UV-B-free light and temperature is desired for industrialized seedling production^72^; CsBRC1, a suppressor of branch outgrowth, is useful for cucumber varieties for fresh fruit production^62^; and the early flowering caused by ‘short-1’ and ‘short-2’ in the upstream region of *CsFT* is advantageous for early marketing and extended harvest^83^.

**Table 1:** Details of identified genes for related shoot architecture traits in cucumber

No.	Mutant or transgenic lines	Phenotype description	Gene name	Location	Candidate gene ID	Gene annotation	References
1	*determinate (de)*	Determinate growth habit	*CsTFL1*	Chr6: 21554312.. 21555486 (-)	*Csa6G452100*	Terminal flower 1a; Phosphatidylethanolamine-binding protein PEBP	Wen et al.^16^
2	*CsLFY-RNAi lines*	Determinate growth habit	*CsLFY*	Chr1: 30604.. 32551 (+)	*Csa1G000050*	LEAFY; Floricaula/leafy protein	Zhao et al.^15^
3	*round leaf 1 (rl-1)*	Round leaves	*CsPID*	Chr1: 19388569.. 19390841 (-)	*Csa1G537400*	Protein kinase	Zhang et al.^38^
4	*round leaf 2 (rl-2)*	Round leaves	*CsPID*	Chr1: 19388569.. 19390841 (-)	*Csa1G537400*	Protein kinase	Zhang et al.^38^
5	*round leaf 3 (rl-3)*	Round leaves	*CsPID*	Chr1: 19388569.. 19390841 (-)	*Csa1G537400*	Protein kinase	Song et al.^36^
6	*round leaf 4 (rl-4)*	Round leaves, and less lateral organs such as tendrils and flowers	*CsPID*	Chr1: 19388569.. 19390841 (-)	*Csa1G537400*	Protein kinase	Liu et al.^37^
7	*mango fruit (mf)*	Butterfly-shaped leaves	*CsWOX1*	Chr1: 4494646.. 4497727 (-)	*Csa1G042780*	WUSCHEL-related homeobox	Niu et al.^40^; Wang et al.^41^
8	*CsIVP-RNAi lines*	Downwardly curled leaf with abnormal leaf veins, compact shoot architecture	*CsIVP*	Chr6: 22272670.. 22274639 (-)	*Csa6G483450*	Transcription factor, basic helix-loop-helix (bHLH) family	Yan et al.^42^
9	*CsYAB5-RNAi lines*	Leaf morphology with overlapped bilateral leaf margins, compact shoot architecture	*CsYAB5*	Chr2: 1216913.. 1220326 (-)	*Csa2G006820*	YABBY protein	Yan et al.^42^
10	*curly leaf-1 (cul-1)*	Upwardly rolled leaf	*CsPHB*	Chr6: 28490993.. 28496793 (-)	*Csa6G525430*	Class III homeobox-leucine zipper protein	Rong et al.^43^
11	*curly leaf-2 (cul-2)*	Upwardly rolled leaf	*CsPHB*	Chr6: 28490993.. 28496793 (-)	*Csa6G525430*	Class III homeobox-leucine zipper protein	Rong et al.^43^
12	*little leaf (ll)*	Little leaf	*CsSAP*	Chr6: 7662821.. 7665390 (-)	*Csa6G111910*	Pentatricopeptide repeat-containing protein	Yang et al.^48^
13	*small and cordate leaf 1 (scl1)*	Small leaf size and cordate leaf with a blunt leaf base	*CsSL1*	Chr7: 3595111.. 3599150 (+)	*Csa7G062760*	PHP domain-containing protein	Gao et al.^49^
14	*CsHAN1-OE lines*	Highly lobed leaves	*CsHAN1*	Chr4: 3624324.. 3626040 (+)	*Csa4G046650*	GATA transcription factor	Ding et al.^50^
15	*CsHAN1-RNAi lines*	Highly lobed leaves	*CsHAN1*	Chr4: 3624324.. 3626040 (+)	*Csa4G046650*	GATA transcription factor	Ding et al.^50^
16	*inbred line S06*	With strong lateral branch growth potential and late lateral branch sprouting time	*CLS*	Chr3: 2859529.. 2861372 (-)	*Csa3G039300*	Transcription factor GRAS; Protein SCARECROW;	Yuan et al.^54^
17	*CsBRC1-RNAi lines*	Increased bud outgrowth	*CsBRC1*	Chr1: 2221849.. 2222877 (+)	*Csa1G020890*	Transcription factor CYCLOIDEA	Shen et al.^62^
18	*CsBRC1p-CsPIN3 lines*	More lateral branches	*CsPIN3*	Chr5: 20035388.. 20039599 (-)	*Csa5G576590*	Auxin efflux carrier	Shen et al.^62^
19	*tendril-less (ten) /ten-1*	Modified tendrils like a branch-like structure	*CsTEN*	Chr5: 27044242.. 27045612 (+)	*Csa5G644520*	a PROLIFERATING CELL FACTORS (TCP) family of transcription factor	Wang et al.^66^
20	*ten-2*	A complete transformation of its tendrils into lateral branches	*CsTEN*	Chr5: 27044242.. 27045612 (+)	*Csa5G644520*	a PROLIFERATING CELL FACTORS (TCP) family of transcription factor	Yang et al.^68^
21	*ten-3*	Some tendrils are most of the normal morphology, while others display slight morphological defect with axillary meristems; both of the tendrils produce a reduced number of helical	*CsTEN*	Chr5: 27044242.. 27045612 (+)	*Csa5G644520*	a PROLIFERATING CELL FACTORS (TCP) family of transcription factor	Yang et al.^68^
22	*ten-4*	Some tendrils are most of the normal morphology, while others display slight morphological defect; both of the tendrils produce a reduced number of helical	*CsTEN*	Chr5: 27044242.. 27045612 (+)	*Csa5G644520*	a PROLIFERATING CELL FACTORS (TCP) family of transcription factor	Yang et al.^68^
23	*aco-1*	The tendril coiling capacity was substantially altered	*CsACO1*	Chr6: 11163849.. 11165376 (+)	*Csa6G160180*	1-Aminocyclopropane-1-carboxylate oxidase	Yang et al.^68^
24	*aco-2*	The tendril coiling capacity was substantially altered	*CsACO1*	Chr6: 11163849.. 11165376 (+)	*Csa6G160180*	1-Aminocyclopropane-1-carboxylate oxidase	Yang et al.^68^
25	*tendril-less1 (td-1)*	A dwarf shoot without tendrils and with less trichome	*CsGCN5*	Chr6: 28883361.. 28894330 (-)	*Csa6G527060*	Histone acetyltransferase gcn5	Chen et al.^70^
26	*short hypocotyl 1 (sh1)*	Hypocotyl elongation is insensitive to UV-B	*CsSH1*	Chr3: 9286025.. 9291984 (+)	*Csa3G141820*	DNA repair helicase rad5,16, putative	Bo et al.^72^
27	*CsGPA1-OE lines*	Enhanced earlier seedling development including hypocotyl elongation	*CsGPA1*	Chr4: 22234946.. 22240824 (-)	*Csa4G648550*	Guanine nucleotide-binding protein alpha-1 subunit	Yan et al.^76^
28	*CsGPA1-RNAi lines*	Inhibited seedling growth	*CsGPA1*	Chr4: 22234946.. 22240824 (-)	*Csa4G648550*	Guanine nucleotide-binding protein alpha-1 subunit	Yan et al.^76^
29	*Cucumber dwarf (Csdw)*	A dwarf phenotype with a reduced internode length	*CsCLV1*	Chr3: 36489540.. 36494263 (-)	*Csa3G872760*	Receptor protein kinase-like protein	Xu et al.^78^
30	*compact (cp)*	An extreme-dwarf-type plant with reduced internode length	*CsCKX*	Chr4: 22175499.. 22179648 (+)	*Csa4G647490*	Cytokinin oxidase/dehydrogenase 1	Li et al.^77^
31	*compact-1 (cp-1)*	A compact growth phenotype	*CsCullin1*	Chr6: 12674524.. 12680892 (+)	*Csa6G197230*	cullin-1 protein	Van der Linden^82^
32	*super compact-1 (scp-1)*	Little hypocotyls elongation and drastically reduced internode length	*CsCYP85A1*	Chr5: 26718684.. 26722145 (+)	*Csa5G641590*	Cytochrome P450; BR-C6-oxidase	Wang et al.^80^
33	*super compact-2 (scp-2)*	Super compact shoot architecture with dark green, wrinkle leaves	*CsDET2*	Chr3: 27762276.. 27763359 (+)	*Csa3G732550*	3-Oxo-5-alpha-steroid 4-dehydrogenase	Hou et al.^81^
34	*short internode (si)*	A dwarf phenotype with a reduced internode length	*CsVFB1*	Chr4: 21396485.. 21399329 (-)	*Csa4G641640*	Putative F-box/LRR-repeat protein 8	Lin et al.^78^
35	*‘long’ UR*	Late flowering time	*CsFT*	Chr1: 25850971.. 25855507 (-)	*Csa1G651710*	Flowering locus T-like 2; Phosphatidylethanolamine-binding protein PEBP	Wang et al.^83^
36	*‘short-1’ UR*	Earlier flowering time	*CsFT*	Chr1: 25850971.. 25855507 (-)	*Csa1G651710*	Flowering locus T-like 2; Phosphatidylethanolamine-binding protein PEBP	Wang et al.^83^
37	*‘short-2’ UR*	Earlier flowering time	*CsFT*	Chr1: 25850971.. 25855507 (-)	*Csa1G651710*	Flowering locus T-like 2; Phosphatidylethanolamine-binding protein PEBP	Wang et al.^83^

Although a large number of plant architecture-related genes have been reported to benefit from rapid advances in techniques in the last five years, some important architectural traits have not yet been investigated in cucumber. For example, leaf angle and lateral branch angle, which greatly affect planting density and crop yield per unit area, await further studies in cucumber. A relatively small leaf angle can improve the accumulation of photosynthetic products by decreasing the amount of mutual shading to capture light for photosynthesis under dense planting, and several genes, such as *Related to ABI3/VP1-Like 1* (*ZmRAVL1*) and *BRASSINOSTEROID-RESPONSIVE LEAF ANGLE REGULATOR 1* (*OsBLR1*), have been identified to play essential roles in this trait in both maize and rice^86,87^. The leaf angle of cucumber plants is even more complex than that of maize and rice, and it is coordinately determined by the angles among the leaf blade, petiole, and stem. Similarly, lateral branch (tiller) angle was shown to directly affect planting density and crop yields, and genes such as *PROSTRATE GROWTH 1* (*PROG1*) and *LAZY1* (*LA1*) are the key players of this trait in cereal crop species^88,89^. Nonetheless, functions of the above homologous genes in cucumber remain elusive and deserve further exploration.

Cucumber is planted all over the world, with several variations in cultivation methods, including open field or greenhouse production, manual harvesting or mechanical harvesting, and productions of fruits for fresh markets or processed pickling. Therefore, the requirements for ideal shoot architecture are different depending on the cultivation method. For cucumber plants cultivated in protected environments for fresh markets, architectural traits such as an indeterminate growth habit, no branching, no tendrils, strong main stems, and small leaf angles are desired. For cucumber plants grown in the open field for processing, moderate determinate habits and branching, compact shoots, and no tendril are advantageous traits composing the ideal shoot architecture.

Future work will not only continue to explore the genes and regulatory mechanisms underlying architectural traits but also identify and integrate appropriate gene resources for fine breeding. An increasing number of bioinformatics and transgene-free techniques based on CRISPR provide the possibility to achieve this goal. With cucumber serving as the model plant species of cucurbits, the genome sequence information of four varieties has been released and updated: the North China spiny-type Chinese Long, the North American pickling cucumber *Gy14*, the wild cucumber Hardwickii (http://cucurbitgenomics.org/), and the North European B10 line (GenBank No. LKUO00000000)^7,90–92^. In addition, deep resequencing of 115 cucumber lines provides a genetic basis of cucumber domestication and diversity^7^. Such progress has facilitated the identification of suitable genes to generate ideal shoot architecture in cucumber. Furthermore, the development of sequencing tools such as RNA-seq, ChIP-seq, and methylation-seq plays a vital role in the study of molecular modulation mechanisms^93^. The CRISPR-Cas9 system, currently the premier genome-editing technique, has been shown to have great application value in the breeding of several crop species, including rice, maize, and tomato^94–96^. CRISPR-Cas9 can be used to edit coding DNA sequences, *cis*-regulatory regions, or other sites influencing gene expression to integrate multiple favorable traits and greatly shorten the breeding process^97^. Moreover, transgene-free plants can be obtained via CRISPR-Cas9 through self-crossing T0 transgenic lines, which greatly increases the possibility of gene-edited vegetables entering the market in the future^98^. De novo domestication has been proposed in tomato^96^, and CRISPR-Cas9 has been used successfully in cucumber^99^, which provides the possibility for rapidly breeding cucumber varieties with an ‘ideal plant type’ for different cultivation methods in the future.

## References

[1] Wang B, Smith SM, Li J (2018). Genetic regulation of shoot architecture. Annu. Rev. Plant Biol..

[2] Mayer KF (1998). Role of *WUSCHEL* in regulating stem cell fate in the Arabidopsis shoot meristem. Cell.

[3] Schoof H (2000). The stem cell population of Arabidopsis shoot meristems in maintained by a regulatory loop between the CLAVATA and WUSCHEL genes. Cell.

[4] Benlloch R (2015). Genetic control of inflorescence architecture in legumes. Front. Plant Sci..

[5] Weberling, F. & Pankhurst. R. J. *Morphology of Flowers and Inflorescences* (Cambridge University Press, Cambridge, 1989).

[6] Prusinkiewicz P, Erasmus Y, Lane B, Harder LD, Coen E (2007). Evolution and development of inflorescence architectures. Science.

[7] Qi J (2013). A genomic variation map provides insights into the genetic basis of cucumber domestication and diversity. Nat. Genet..

[8] Leonard ER (1962). Inter-relations of vegetative and reproductive growth, with special reference to indeterminate plants. Bot. Rev..

[9] Weng Y, Johnson S, Staub JE, Huang S (2010). An extended intervarietal microsatellite linkage map of cucumber, *Cucumis sativus* L. Hort. Science.

[10] Doebley J (2004). The genetics of maize evolution. Annu. Rev. Genet..

[11] Park SJ (2014). Optimization of crop productivity in tomato using induced mutations in the florigen pathway. Nat. Genet..

[12] Paran I, van der Knaap E (2007). Genetic and molecular regulation of fruit and plant domestication traits in tomato and pepper. J. Exp. Bot..

[13] Spielmeyer W, Ellis MH, Chandler PM (2002). Semidwarf (*sd-1*), “green revolution” rice, contains a defective gibberellin 20-oxidase gene. Proc. Natl Acad. Sci. USA.

[14] Wang Y, Li J (2008). Molecular basis of plant architecture. Annu. Rev. Plant Biol..

[15] Zhao W (2018). *CsLFY* is required for shoot meristem maintenance via interaction with WUSCHEL in cucumber (*Cucumis sativus*). N. Phytol..

[16] Wen C (2019). CsTFL1 inhibits determinate growth and terminal flower formation through interaction with CsNOT2a in cucumber. Development.

[17] George WL (1970). Genetic and environmental modification of determinate plant habit in cucumbers. J. Am. Soc. Hortic. Sci..

[18] Miller GA, George WL (1979). Inheritance of dwarf and determinate growth habits in cucumber. J. Am. Soc. Hortic. Sci..

[19] Liljegren SJ, Gustafson-Brown C, Pinyopich A, Ditta GS, Yanofsky MF (1999). Interactions among APETALA1, LEAFY, and TERMINAL FLOWER1 specify meristem fate. Plant Cell.

[20] Gustafson-Brown C, Savidge B, Yanofsky MF (1994). Regulation of the Arabidopsis floral homeotic gene APETALA1. Cell.

[21] Goslin K (2017). Transcription factor Interplay between LEAFY and APETALA1/CAULIFLOWER during Floral Initiation. Plant Physiol..

[22] Hanano S, Goto K (2011). Arabidopsis *TERMINAL FLOWER1* is involved in the regulation of flowering time and inflorescence development through transcriptional repression. Plant Cell.

[23] Bowman JL, Alvarez J, Weigel D, Meyerowitz EM, Smyth DR (1993). Control of flower development in *Arabidopsis thaliana* by *APETALA1* and interacting genes. Development.

[24] Alvarez J, Guli CL, Yu XH, Smyth DR (1992). Terminal flower: a gene affecting inflorescence development in *Arabidopsis thaliana*. Plant J..

[25] Bradley D, Ratcliffe O, Vincent C, Carpenter R, Coen E (1997). Inflorescence commitment and architecture in Arabidopsis. Science.

[26] Blazquez MA, Ferrandiz C, Madueño F, Parcy F (2006). How floral meristems are built. Plant Mol. Biol..

[27] Silva WB (2018). SELF-PRUNING acts synergistically with DIAGEOTROPICA to guide auxin responses and proper growth form. Plant Physiol..

[28] Bradley D, Vincent C, Carpenter R, Coen E (1996). Pathways for inflorescence and floral induction in Antirrhinum. Development.

[29] Nakagawa M, Shimamoto K, Kyozuka J (2002). Overexpression of RCN1 and RCN2, rice TERMINAL FLOWER 1/CENTRORADIALIS homologs, confers delay of phase transition and altered panicle morphology in rice. Plant J..

[30] Lee C (2019). Genetic interactions reveal the antagonistic roles of FT/TSF and TFL1 in the determination of inflorescence meristem identity in Arabidopsis. Plant J..

[31] Abe M (2005). FD, a bZIP protein mediating signals from the floral pathway integrator FT at the shoot apex. Science.

[32] Jiang K, Liberatore KL, Park SJ, Alvarez JP, Lippman ZB (2013). Tomato yield heterosis is triggered by a dosage sensitivity of the florigen pathway that fine-tunes shoot architecture. PLoS Genet..

[33] Krieger U, Lippman ZB, Zamir D (2010). The flowering gene SINGLE FLOWER TRUSS drives heterosis for yield in tomato. Nat. Genet..

[34] Du F, Guan C, Jiao Y (2018). Molecular mechanisms of leaf morphogenesis. Mol. Plant.

[35] Ichihashi Y (2011). Key proliferative activity in the junction between the leaf blade and leaf petiole of Arabidopsis. Plant Physiol..

[36] Song M (2019). A leaf shape mutant provides insight into PINOID serine/threonine kinase function in cucumber (*Cucumis sativus* L.). J. Integr. Plant Biol..

[37] Liu X (2019). PINOID is required for lateral organ morphogenesis and ovule development in cucumber. J. Exp. Bot..

[38] Zhang CW (2018). Mutations in *CsPID* encoding a Ser/Thr protein kinase are responsible for round leaf shape in cucumber (*Cucumis sativus* L.). Theor. Appl. Genet..

[39] Benjamins R, Quint A, Weijers D, Hooykaas P, Offringa R (2001). The PINOID protein kinase regulates organ development in Arabidopsis by enhancing polar auxin transport. Development.

[40] Niu H (2018). The WUSCHEL-related homeobox1 gene of cucumber regulates reproductive organ development. J. Exp. Bot..

[41] Wang H (2020). WUSCHEL-related homeobox1 (WOX1) regulates vein patterning and leaf size in *Cucumis sativus*. Hortic. Res..

[42] Yan S (2020). CsIVP functions in vasculature development and downy mildew resistance in cucumber. PLoS Biol..

[43] Rong F (2019). A mutation in class III homeodomain‑leucine zipper (HD‑ZIP III) transcription factor results in *curly leaf* (*cul*) in cucumber (*Cucumis sativus* L.). Theor. Appl. Genet..

[44] McConnell JR (2001). Role of PHABULOSA and PHAVOLUTA in determining radial patterning in shoots. Nature.

[45] Emery JF (2003). Radial patterning of Arabidopsis shoots by class III HD-ZIP and KANADI genes. Curr. Biol..

[46] McConnell JR, Barton MK (1998). Leaf polarity and meristem formation in Arabidopsis. Development.

[47] Czesnick H, Lenhard M (2015). Size control in plants-lessons from leaves and flowers. Cold Spring Harb. Perspect. Biol..

[48] Yang L (2018). *LITTLELEAF* (*LL*) encodes a WD40 repeat domain-containing protein associated with organ size variation in cucumber. Plant J..

[49] Gao D (2017). Mutation in a novel gene *SMALL AND CORDATE LEAF 1* affects leaf morphology in cucumber. J. Integr. Plant Biol..

[50] Ding L (2015). HANABA TARANU regulates the shoot apical meristem and leaf development in cucumber (*Cucumis sativus* L.). J. Exp. Bot..

[51] Schumacher K, Schmitt T, Rossberg M, Schmitz G, Theres K (1999). The *Lateral suppressor* (*Ls*) gene of tomato encodes a new member of the VHIID protein family. Proc. Natl Acad. Sci. USA.

[52] Yang F, Wang Q, Schmitz G, Muller D, Theres K (2012). The bHLH protein ROX acts in concert with RAX1 and LAS to modulate axillary meristem formation in Arabidopsis. Plant J..

[53] Li X (2003). Control of tillering in rice. Nature.

[54] Yuan L (2010). The *Cucumber Lateral Suppressor Gene* (*CLS*) is functionally associated with axillary meristem initiation. Plant Mol. Biol. Rep..

[55] De Smet I, Jurgens G (2007). Patterning the axis in plants-auxin in control. Curr. Opin. Genet. Dev..

[56] Domagalska MA, Leyser O (2011). Signal integration in the control of shoot branching. Nat. Rev. Mol. Cell Biol..

[57] Aguilar-Martínez JA, Poza-Carrión C, Cubas P (2007). Arabidopsis BRANCHED1 acts as an integrator of branching signals within axillary buds. Plant Cell.

[58] Martín-Trillo M (2011). Role of tomato BRANCHED1-like genes in the control of shoot branching. Plant J..

[59] Finlayson SA (2007). Arabidopsis Teosinte Branched1-like 1 regulates axillary bud outgrowth and is homologous to monocot Teosinte Branched1. Plant Cell Physiol..

[60] Doebley J, Stec A, Gustus C (1995). *teosinte branched1* and the origin of maize: evidence for epistasis and the evolution of dominance. Genetics.

[61] Hubbard L, McSteen P, Doebley J, Hake S (2002). Expression patterns and mutant phenotype of *teosinte branched1* correlate with growth suppression in maize and teosinte. Genetics.

[62] Shen J (2019). CsBRC1 inhibits axillary bud outgrowth by directly repressing the auxin efflux carrier CsPIN3 in cucumber. Proc. Natl Acad. Sci. USA.

[63] Kiss JZ (2006). Up, down, and all around: how plants sense and respond to environmental stimuli. Proc. Natl Acad. Sci. USA.

[64] Isnard S, Silk WK (2009). Moving with climbing plants from Charles Darwin’s time into the 21st century. Am. J. Bot..

[65] Sousa-Baena MS, Lohmann LG, Hernandes-Lopes J, Sinha NR (2018). The molecular control of tendril development in angiosperms. N. Phytol..

[66] Wang S (2015). A rare SNP identified a TCP transcription factor essential for tendril development in cucumber. Mol. Plant..

[67] Gerbode SJ, Puzey JR, McCormick AG, Mahadevan L (2012). How the cucumber tendril coils and overwinds. Science.

[68] Yang X (2020). Regulation of plant architecture by a new histone acetyltransferase targeting gene bodies. Nat. Plants.

[69] Mizuno S (2015). Chiba tendril-less locus determines tendril organ identity in melon (*Cucumis melo* L.) and potentially encodes a tendril-specific TCP homolog. J. Plant Res..

[70] Chen F (2017). Fine mapping identifies *CsGCN5* encoding a histone acetyltransferase as putative candidate gene for *tendril‑less1* mutation (*td‑1*) in cucumber. Theor. Appl. Genet..

[71] Huang Y, Kong Q, Chen F, Bie Z (2015). The history, current status and future prospects of vegetable grafting in China. Acta Hortic..

[72] Bo K (2016). *SHORT HYPOCOTYL1* encodes a SMARCA3-like chromatin remodeling factor regulating elongation. Plant Physiol..

[73] López-Juez E (1992). The cucumber long hypocotyl mutant lacks a light-stable PHYB-like phytochrome. Plant Cell.

[74] De Lucas M (2008). A molecular framework for light and gibberellin control of cell elongation. Nature.

[75] Liu B (2021). Plant buffering against the high-light stress induced accumulation of *CsGA2ox8* transcripts via alternative splicing to finely tune gibberellin levels and maintain hypocotyl elongation. Hortic. Res..

[76] Yan Y (2018). Functions of CsGPA1 on the hypocotyl elongation and root growth of cucumbers. Sci. Rep..

[77] Li Y (2011). Fine genetic mapping of *cp*: a recessive gene for compact (dwarf) plant architecture in cucumber, *Cucumis sativus* L. Theor. Appl. Genet..

[78] Xu L, Wang C, Cao W, Zhou S, Wu T (2018). CLAVATA1-type receptor-like kinase CsCLAVATA1 is a putative candidate gene for *dwarf* mutation in cucumber. Mol. Genet. Genomics.

[79] Lin T (2016). A truncated F-Box protein confers the dwarfism in cucumber. J. Genet. Genomics.

[80] Wang H (2017). The cytochrome P450 Gene *CsCYP85A1* is a putative candidate for *Super Compact-1* (*Scp-1*) plant architecture mutation in cucumber (*Cucumis sativus* L.). Front. Plant Sci..

[81] Hou S (2017). A mutant in the *CsDET2* gene leads to a systemic brassinosteriod deficiency and super compact phenotype in cucumber (*Cucumis sativus* L.). Theor. Appl. Genet..

[82] Van der Linden, L. Marker for compact growth in cucumber. Patent WO/2017/042272 (2018).

[83] Wang S (2020). *FLOWERING LOCUS T* improves cucumber adaptation to higher latitudes. Plant Physiol..

[84] Pawełkowicz M, Skarzyńska A, Pląder W, Przybecki Z (2019). Genetic and molecular bases of cucumber (*Cucumis sativus* L.) sex determination. Mol. Breed..

[85] Che Q, Zhang X (2019). Molecular basis of cucumber fruit domestication. Curr. Opin. Plant Biol..

[86] Tian J (2019). Teosinte ligule allele narrows plant architecture and enhances high-density maize yields. Science.

[87] Wang K (2020). The basic helix-loop-helix transcription factor OsBLR1 regulates leaf angle in rice via brassinosteroid signalling. Plant Mol. Biol..

[88] Wu Y (2018). Deletions linked to PROG1 gene participate in plant architecture domestication in Asian and African rice. Nat. Commun..

[89] Yoshihara T, Iino M (2007). Identification of the gravitropism-related rice gene LAZY1 and elucidation of LAZY1-dependent and -independent gravity signaling pathways. Plant Cell Physiol..

[90] Yang L (2012). Chromosome rearrangements during domestication of cucumber as revealed by high-density genetic mapping and draft genome assembly. Plant J..

[91] Li Q (2019). A chromosome-scale genome assembly of cucumber (*Cucumis sativus* L.). Gigascience.

[92] Osipowski P (2020). A high-quality cucumber genome assembly enhances computational comparative genomics. Mol. Genet. Genomics.

[93] Muhammad II, Kong SL, Akmar Abdullah SN, Munusamy U (2019). RNA-seq and ChIP-seq as complementary approaches for comprehension of plant transcriptional regulatory mechanism. Int. J. Mol. Sci..

[94] Toda E (2019). An efficient DNA- and selectable-marker-free genome-editing system using zygotes in rice. Nat. Plants.

[95] Agarwal A (2018). Insights into maize genome editing via CRISPR/Cas9. Physiol. Mol. Biol. Plants.

[96] Zsögön A (2018). *De novo* domestication of wild tomato using genome editing. Nat. Biotechnol..

[97] Li T (2018). Domestication of wild tomato is accelerated by genome editing. Nat. Biotechnol..

[98] Rodríguez-Leal D, Lemmon ZH, Man J, Bartlett ME, Lippman ZB (2017). Engineering quantitative trait variation for crop improvement by genome editing. Cell.

[99] Hu B (2017). Engineering non-transgenic gynoecious cucumber using an improved transformation protocol and optimized CRISPR/Cas9 system. Mol. Plant.

